# Development and Test of a Novel High-Precision Inchworm Piezoelectric Motor

**DOI:** 10.3390/mi16090992

**Published:** 2025-08-29

**Authors:** Nan Huang, Jiahao Yin, Fuyuan Feng, Lanyu Zhang, Yuheng Luo, Jian Gao

**Affiliations:** State Key Laboratory of Precision Electronic Manufacturing Technology and Equipment, Guangdong University of Technology, Guangzhou 510006, China; 12432378@mail.sustech.edu.cn (N.H.); 3122005452@mail2.gdut.edu.cn (J.Y.); 1112401031@mail2.gdut.edu.cn (F.F.); 1112101004@mail2.gdut.edu.cn (Y.L.); gaojian@gdut.edu.cn (J.G.)

**Keywords:** precision motion, piezoelectric actuation, flexible mechanism, motion stage

## Abstract

The inchworm piezoelectric motor, with the advantages of long stroke and high resolution, is ideally suited for precise positioning in wafer-level electron beam inspection systems. However, the large number of piezoelectric actuators and the complex excitation signal sequences significantly increase the complexity of system assembly and temporal control. A flexure-based actuation stator structure, along with simplified excitation signal sequences of a high-precision inchworm piezoelectric motor, is proposed. The alternating actuation of upper/lower clamping mechanisms and the driving mechanism fundamentally mitigates backstep effects while generating stepping linear displacement. The inchworm piezoelectric motor achieves precision linear motion operation using only two piezoelectric actuators. The actuation stator is analyzed via the compliance matrix method to derive its output compliance, input stiffness, and displacement amplification ratio. Furthermore, a kinematic model and natural frequency expression incorporating the pseudo-rigid-body method and Lagrange’s equations are established. The actuation stator and inchworm piezoelectric motor are analyzed through both simulations and experiments. The results show that the maximum step displacement of the motor is 16.3 μm, and the maximum speed is 9.78 mm/s, at a 600 Hz operation frequency with a combined alternating piezoelectric voltage of 135 V and 65 V. These findings validate the designed piezoelectric motor’s superior motion resolution, operational stability, and acceptable load capacity.

## 1. Introduction

Precision actuators play a critical role in precision positioning and motion operations [1,2,3,4]. With the advancement of electronic manufacturing and inspection technologies, the requirements for precision and resolution in wafer-level electron beam inspection systems have become increasingly stringent. Traditional actuators, such as three-phase motors and stepper motors, offer sufficient power and advantages like high stiffness and load capacity [5,6,7]. However, traditional actuators inherently rely on relative motion between components, leading to limitations, such as friction, wear, and fatigue, which restrict positioning accuracy. These limitations are increasingly prompting the adoption of novel actuator technologies that meet the growing demand for high-precision positioning and speed [8,9,10].

Piezoelectric-driven nano-positioning technology, as a novel driving method, utilizes the inverse piezoelectric effect of piezoelectric materials to convert electrical energy into mechanical energy, achieving motion output through friction between the stator and rotor. It has been widely adopted in precision engineering fields due to its superior advantages, including high displacement resolution, immunity to electromagnetic interference, and rapid response speed [11,12,13,14]. Piezoelectric motor types include ultrasonic type [15,16,17], inertial type [18,19,20,21], and inchworm type [22,23,24]. Direct-driven piezoelectric actuators can achieve high-precision positioning by using the micro-deformation of flexible amplification structures, but their driving stroke is still limited by the mechanism [25]. As a category of piezoelectric actuators, the inchworm-type piezoelectric motor imitates the inchworm’s creeping motion, accumulating micro-displacements in each motion cycle to achieve stepping motion. The inchworm piezoelectric motor exhibits an unlimited stroke and high repeatability, making it ideally suited for wafer-level electron beam inspection systems [22].

Due to the unique advantages of the inchworm-type piezoelectric motor, many studies have been conducted in recent years, which have greatly promoted its applications. Dong et al. proposed an inchworm actuator that employs a compound mechanism of a lever amplifier and a bridge amplifier. Motion is realized through the cooperation of the amplification mechanism and an elastomer [26]. Ling et al. proposed an improved pusher-type structure with simplified excitation signal sequences. The experiments found that a large step size under high motion speed can be achieved with the design [27]. Deng et al. proposed a compact inchworm piezoelectric actuator that employs a three-jaw clamping mechanism to simplify the excitation signal sequences. Under periodic control, the clamping jaws achieve multidirectional friction adjustment, which significantly enhances the load capacity and motion stability [28]. Ma et al. proposed an inchworm actuator with a symmetrical clamping mechanism, utilizing two piezoelectric actuators, which effectively suppress backward motion under high loads [29]. Many researchers have extensively studied the operating principle, structure design, and backstep motion of the inchworm piezoelectric motor, which has greatly promoted the development of the inchworm-type piezoelectric motor. However, challenges, such as the number and cost of piezoelectric actuations, complex excitation signal sequences, and significant backstep effects, increase the complexity of precision motion, impeding further applications of piezoelectric motors.

Based on the rapid development requirements of the electronic manufacturing field and the insights gained from the aforementioned studies, a novel high-precision inchworm piezoelectric motor integrating a flexure-based actuation stator and simplified excitation signal sequences is proposed. For the inchworm piezoelectric motor, the actuation stator utilizes two piezoelectric elements. Under the control of the simplified excitation signal sequences, through the displacement amplification mechanisms, the output displacement of the piezoelectric elements is converted into motion of the upper/lower clamping mechanisms and the driving mechanism within the actuation stator. Meanwhile, this inchworm piezoelectric motor can also achieve a stepping linear displacement by mitigating backstep effects. This novel method achieves precision displacement utilizing two piezoelectric elements while significantly reducing assembly complexity and simplifying temporal control requirements. This design provides a novel reference for the structural design and excitation signal sequence control of existing inchworm piezoelectric motors, heralding potential adoption in electronic manufacturing and the inspection field.

The rest of this paper is arranged as follows: Section 2 introduces the configuration and operating principle of the proposed inchworm piezoelectric motor. Section 3 presents the analytical solutions for the output compliance, input stiffness, and displacement amplification ratio of the actuation stator using the compliance matrix method. Furthermore, a kinematic model for the proposed stator is also established. Section 4 evaluates the actuation stator and motion stage through finite element analysis, validating the correctness of the operating principle. Section 5 constructs a dedicated experimental setup for the inchworm piezoelectric motor to test the kinematic/mechanical performance, including displacement, velocity, and load capacity. Finally, Section 6 provides the conclusion of this work.

## 2. Configuration and Operating Principle

### 2.1. Structure of the Motor

**Figure 1:**
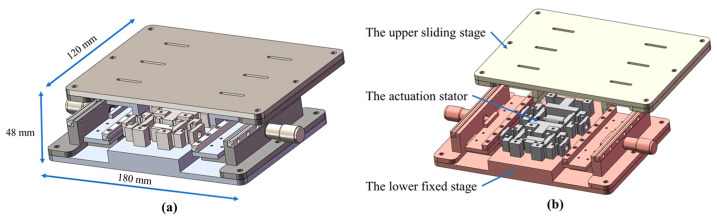
The structure of the inchworm piezoelectric motor: (**a**) inchworm piezoelectric motor; (**b**) overall composition of the motor.

Figure 1a shows the overall structure of the inchworm piezoelectric motor with a total length of 180 mm, a width of 120 mm, and an overall height of 48 mm. As shown in Figure 1b, this motor consists of three parts: a lower fixed stage (the red components), an actuation stator (the gray components), and an upper sliding stage (the light-yellow components). The lower fixed stage is rigidly mounted on the experimental bench while being bolted to the actuation stator and coupled with the upper sliding stage via four sets of precision linear guides to form a sliding pair. The actuation stator integrates dual piezoelectric actuator elements as the core dynamic component of the inchworm piezoelectric motor. The upper sliding stage not only interfaces with the lower fixed stage but also directly contacts the actuation stator.

**Figure 2:**
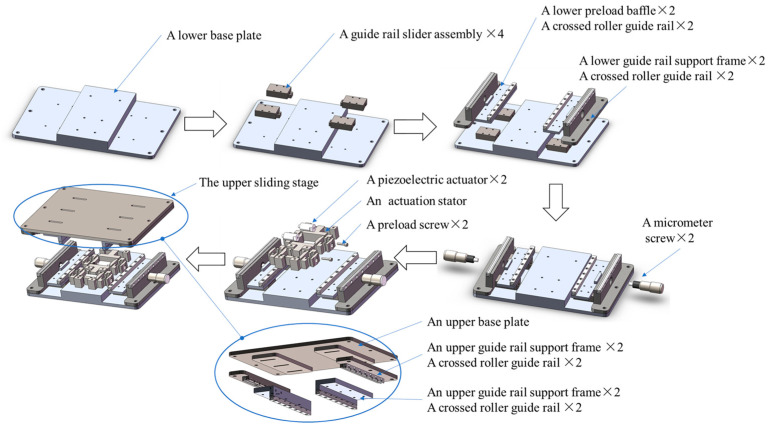
Components and assembly process of the inchworm piezoelectric motor.

As shown in Figure 2, the lower fixed stage includes a lower base plate, four sets of guide rail slider assemblies, two lower preload baffles, four crossed roller guide rails, two lower guide rail support frames, and two micrometer screws.

Compared to the lower fixed stage, the upper sliding stage omits the four sets of guide rail slider assembly and adds the slot structures on the upper base plate for preload baffle adjusting and positioning. The remaining components are similar to the lower fixed stage. It should be noted that structural disassembly reveals that the upper sliding stage establishes contact with the actuation stator via upper preload baffles on both sides, forming a controllable friction interface. Meanwhile, micrometer screws transmit radial forces through the upper/lower preload baffles to the actuation stator, thereby adjusting contact preload between the upper baffles and actuation stator to generate variable friction effects.

### 2.2. Actuation Stator

The actuation stator is fabricated from AL 7075-T651, with its material properties shown in Table 1.

The actuation stator incorporates right-angle flexible hinges for connections. Compared to rigid mechanisms, the flexible hinges offer advantages including compact structure, simplified design, and ease of fabrication [30,31]. Two piezoelectric actuators (PSt150/5/7VS10 XMT) are selected to drive the motion motor. Compared with other actuators, such as shape memory alloys and conventional linear actuators, piezoelectric actuators exhibit ultra-high driving force, nanometer-level motion resolution, and high response speed [32]. However, the effective stroke of piezoelectric actuators is typically limited to under 200 μm, which significantly restricts millimeter-scale applications [33]. To overcome this limitation, the actuation stator in this study employs two-stage displacement amplification mechanisms.

**Figure 3:**
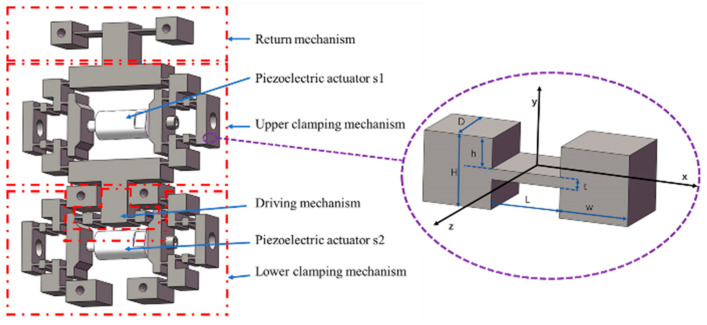
The structure of the actuation stator.

As shown in Figure 3, the actuation stator comprises the following components: the upper/lower clamping mechanisms, the driving mechanism, and the return mechanism. Both the upper and lower clamping mechanisms consist of two symmetric clamping units positioned on the left and right sides. When piezoelectric actuator s1 extends, the upper clamping mechanism amplifies its displacement, clamping the upper preload baffle of the upper sliding stage. Similarly, when piezoelectric actuator s2 extends, the lower clamping mechanism amplifies its displacement and clamps the upper preload baffle. Additionally, the extension of piezoelectric actuator s2 induces forward displacement of the driving mechanism along the positive y-axis. The return mechanism assists in resetting the driving mechanism while avoiding the generation of a cantilever structure.

### 2.3. Operating Principle

**Figure 4:**
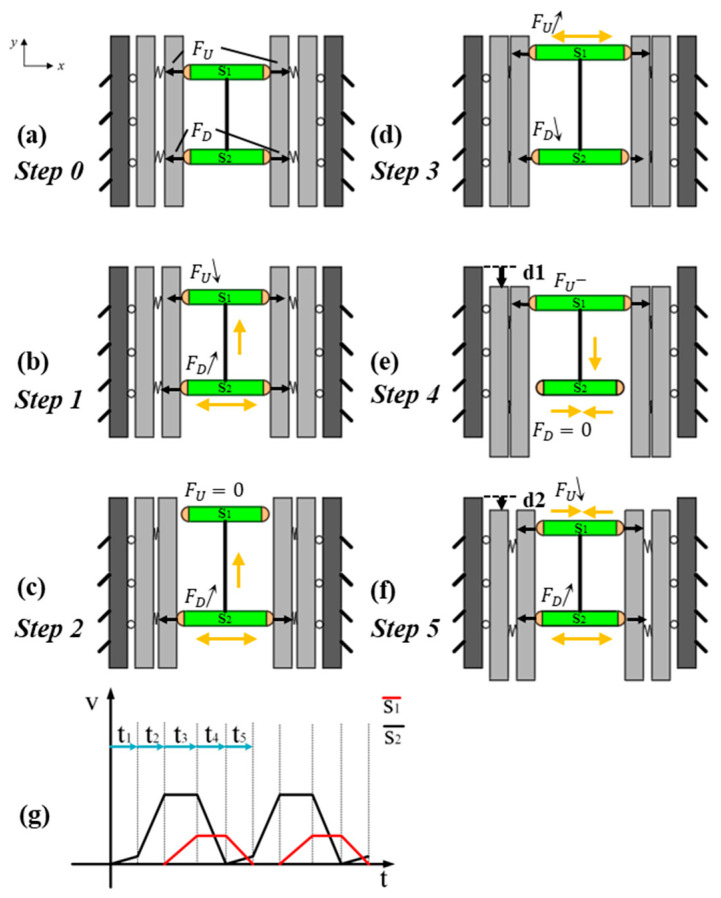
Motion generation strategy. (**a**–**f**) Process of motion generation; (**g**) excitation signal sequence of each actuator.

Based on the proposed piezoelectric motor, the working principle of motion generation that achieves linearity and backstep effect mitigation is presented in Figure 4. In the running process, the upper and lower clamping mechanisms work alternately to enable the upper sliding stage to execute deterministic movement (eliminating random displacement), while the driving mechanism performs periodic motion to ensure the stage generates a periodically stable gait. A specific explanation is demonstrated as follows.

Step 0: Prior to energization, both piezoelectric actuator s1 and piezoelectric actuator s2 remain at their nominal lengths. In this power-off self-locking state, the upper clamping mechanism applies a normal force $F_{U}$ to the upper preload baffle, while the lower clamping mechanism applies an equal normal force $F_{D}$ to the upper preload baffle, satisfying $F_{U}=F_{D},$ as shown in Figure 4a.

Step 1: s2 is energized with a low voltage, causing bidirectional extension at both ends. This results in a minor forward displacement of the driving mechanism along the positive y-axis. Concurrently, the lower clamping mechanism increases its normal force applied to the upper preload baffle, resulting in $F_{D}↑$, $F_{U}{<F}_{D}$, as shown in Figure 4b.

Step 2: Increasing the operating voltage of s2 induces further extension at both ends, which drives the driving mechanism toward the positive y-axis with amplified displacement. At this high-voltage operational state, the driving mechanism reaches its maximum achievable displacement in the positive y-axis direction. Concurrently, the lower clamping mechanism significantly enhances the normal force applied to the upper preload baffle. As shown in Figure 4c, $F_{D}↑$.

Step 3: s2 maintains its voltage and no extension at both ends. s1 is energized to a medium voltage level, causing the upper clamping mechanism to elevate the normal force on the upper preload baffle. As shown in Figure 4d, $F_{U}↑$, $F_{U}{>F}_{D}$.

Step 4: The operating voltage of s2 drops to zero and its ends retract to their original length. This causes the normal pressure exerted by the lower clamping mechanism on the upper preload baffle to decrease, and the driving mechanism retracts to its initial state. Meanwhile, s1 remains energized at before voltage. As shown in Figure 4e, due to the sustained clamping force from the upper clamping mechanism on the upper preload baffle, the upper sliding stage moves with the driving mechanism’s contraction, resulting in a negative y-axis displacement $d_{1}$.

Step 5: The operating voltage of s2 increases to a low level, causing the lower clamping mechanism to elevate the normal force on the upper preload baffle, $F_{D}↑$. This induces a small positive y-axis displacement in the driving mechanism. Meanwhile, the operating voltage of s1 decreases to zero. When the operating voltage of s1 exceeds that of s2, $F_{U}{>F}_{D}$, causing the upper sliding stage to backstep slightly $d_{2}-d_{1}$, as shown in Figure 4f. However, during this phase, the rate of voltage decrease for s1 is significantly greater than the rate of voltage increase for s2, mitigating the backstep effect. Subsequently, the inchworm piezoelectric motor initiates a new cycle.

In the end, the presented inchworm piezoelectric motor can accomplish displacement with linearity and backstep effect mitigation by loop execution of the above steps (from step 1 to step 5). Theoretically, the generated motion can be linearly controlled solely by the voltages of two piezoelectric actuators, significantly reducing control complexity. The excitation signal sequence of each piezoelectric actuator is shown in Figure 4.

## 3. Establishment of the Kinematic Model for the Actuation Stator

### 3.1. Modeling of Output Compliance

The relationship between the displacement generated at the free end of a planar single-axis flexure hinge with the corresponding applied load can be expressed as:

(1) δ=CF.

where $\delta ={\left[\delta_{x},\delta_{y},\delta_{z}\right]}^{T}$, represents the displacements along the x- and y-axes and the rotational angle about the z-axis at the free end of the flexure hinge. $F={\left[F_{x},F_{y},M_{z}\right]}^{T}$ represents the loads applied in the free end. C is the corresponding compliance matrix of the flexure hinge. The compliance matrix of a planar single-axis flexure hinge can be expressed as:

(2) $$C=\left[\begin{array}{ccc} C_{x,x} & 0 & 0\\ 0 & C_{y,y} & C_{y,z}\\ 0 & C_{z,y} & C_{z,z} \end{array}\right],$$

where $C_{m,n}$ represents the compliance of the flexure hinge along the m-direction under a load applied in the n-direction.

The compliance matrix $C_{i}$ of the flexure hinge in coordinate system $O_{i}$ can be transformed to the compliance matrix $C_{j}$ in coordinate system $O_{j}$ through coordinate transformation. The transformation formula is given by:

(3) $$C_{j}=T_{i}^{j}C_{i}{\left(T_{i}^{j}\right)}^{T},$$

where $T_{i}^{j}$ represents a homogeneous coordinate transformation matrix:

(4) $$T_{i}^{j}=\left[\begin{array}{cc} R_{i}^{j} & S(r_{i}^{j})R_{i}^{j}\\ 0 & R_{i}^{j} \end{array}\right].$$

where $R_{i}^{j}$ represents the rotation matrix corresponding to the transformation from coordinate system $O_{i}$ to $O_{j}$.

In a serial flexure hinge mechanism, the output displacement of the end effector is accumulated through the deflection of elastic deformation across the entire flexure hinge. Therefore, assuming the flexure hinge mechanism consists of *k* flexure hinge units connected in series, the compliance $C_{o}$ of the serial mechanism in the global coordinate system O can be derived as:

(5) $$C_{O}=\sum_{i=1}^{k}T_{O_{i}}^{O}C_{i}{\left(T_{O_{i}}^{O}\right)}^{T},$$

where $C_{i}$ represents the compliance of the *i*-th flexure hinge in its local coordinate system.

In a parallel flexure hinge mechanism, assuming the terminal configuration is formed by k flexure hinge mechanisms connected in parallel, the stiffness of the flexible configuration in the global coordinate system O corresponds to the combined stiffness of the k flexure hinge mechanisms. Consequently, the compliance matrix $C_{O}$ can be expressed as:

(6) $$C_{O}={\left(\sum_{i=1}^{k}{\left(T_{O_{i}}^{O}C_{i}{\left(T_{O_{i}}^{O}\right)}^{T}\right)}^{-1}\right)}^{-1}.$$

The coefficients of the compliance matrix for a straight-beam flexure hinge can be estimated using the following empirical formulas [34]:

(7) $$\left\{\begin{array}{c} C_{x,x}=\frac{1}{EDH}\left[4h\times {\left(\frac{h}{t}\right)}^{0.0709}\times {\left(\frac{L}{t}\right)}^{0.7515}+L+2w\right]\\ C_{y,y}=\frac{5.2749}{ED}\times {\left(\frac{h}{t}\right)}^{0.0124}\times {\left(\frac{L}{t}\right)}^{2.8996}+\frac{\alpha E}{G}\\ C_{y,z}=\frac{8.3493}{EDt}\times {\left(\frac{L}{t}\right)}^{1.8842}\\ C_{y,z}\approx C_{z,y}\\ C_{z,z}=\frac{16.5350}{EDt^{2}}\times {\left(\frac{L}{t}\right)}^{0.8856} \end{array}\right.$$

where *D*, *H*, *h*, *t*, *L*, and *w* are geometric parameters of the hinge, as shown in Figure 3.

**Figure 5:**
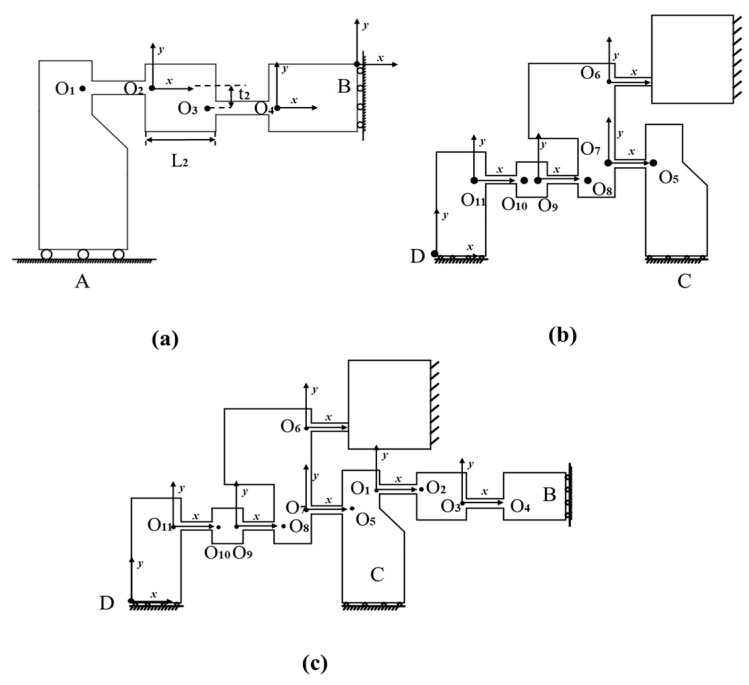
Analytical models of the actuation stator. (**a**) The left half of the driving mechanism. (**b**) The left half of the upper clamping mechanism. (**c**) The left half of the lower clamping mechanism.

In the design of the piezoelectric motor, the driving mechanism is essentially a bridge-type amplification mechanism with a symmetrical left-right structure. Therefore, to derive the output compliance model of the driving mechanism, the left half is selected as the research object, and its detailed coordinate system is shown in Figure 5a.

Based on (5) and (6), the output compliance formula for the left half of the driving mechanism can be derived as:

(8) CABl=T2BC2(T2B)T+T4BC4(T4B)T.

The output compliance formula for the right half can be derived by rotating 180 degrees about the y-axis:

(9) CABr=(Tlr)CABl(Tlr)T.

To sum up, the output compliance formula for the driving mechanism can be derived as:

(10) CB=((CABl)−1+(CABr)−1)−1.

To derive the output compliance model of the upper clamping mechanism, the left section is analyzed as the research object, with its detailed coordinate system shown in Figure 5b.

Based on (5) and (6), the output compliance formula for the left-upper half of the clamping mechanism can be derived as:

(11) CCDuu=T8D((T68C6(T68)T)−1+(T78C7(T78)T)−1)−1T8D+T9DC9(T9D)T+T11DC11(T11D)T.

The output compliance formula for the upper-right half can be derived by rotating 180 degrees about the x-axis:

(12) CCDud=(Tud)CCDuu(Tud)−1.

To sum up, the output compliance formula for the upper clamping mechanism can be derived as:

(13) CDu=((CCDuu)−1+(CCDud)−1)−1.

To derive the output compliance model of the lower clamping mechanism, the lower-left section is analyzed as the research object. Compared to the upper clamping mechanism, the lower clamping mechanism is connected in series with two bridge-type amplification mechanisms, as shown in Figure 5c. Based on (5) and (6), The output compliance of the lower clamping mechanism can be derived as:

(14) CCDuu=T8D((T68C6(T68)T)−1+(T78C7(T78)T+T18C1(T18)T+T38C3(T38)T)−1)−1T8D+ T9DC9(T9D)T+T11DC11(T11D)T,

(15) CCDdd=(Tud)CCDdu(Tud)−1,

(16) CDd=((CCDdu)−1+(CCDdd)−1)−1.

### 3.2. Modeling of Input Stiffness

For the driving mechanism, the input stiffness can be derived as:

(17) $$K_{A}={\left(T_{3}^{A}C_{3}{\left(T_{3}^{A}\right)}^{T}+T_{1}^{A}C_{1}{\left(T_{1}^{A}\right)}^{T}\right)}^{-1}+{\left(T_{u}^{d}{\left(T_{3}^{A}C_{3}{\left(T_{3}^{A}\right)}^{T}+T_{1}^{A}C_{1}{\left(T_{1}^{A}\right)}^{T})(T_{u}^{d}\right)}^{T}\right)}^{-1}.$$

For the upper clamping mechanism, the input stiffness can be derived as:

(18) KDu=(T7C((T107C10(T107)T+T87C8(T87)T)−1+(T67C6(T67)T)−1)−1(T7C)T+T5CC5(T5C)T)−1.

For the lower clamping mechanism, the input stiffness can be derived as:

(19) KDd=(T7C((T107C10(T107)T+T87C8(T87)T)−1+(T67C6(T67)T)−1)−1(T7C)T+T5CC5(T5C)T)−1+(T3CC3(T3C)T+T1CC1(T1C)T)−1.

### 3.3. Mechanical Amplification Ratio

For the driving mechanism, the displacement amplification ratio can be derived as [34]:

(20) $$R_{B}=\frac{3t_{2}(L+3L_{2})}{t^{2}+9{t_{2}}^{2}}.$$

For the upper/lower clamping mechanism, both exhibit the same displacement amplification ratio, which can be derived by simplifying to a lever model:

(21) CDu=CDd=l2l1.

### 3.4. Development and Analysis of the Kinematic Model

**Figure 6:**
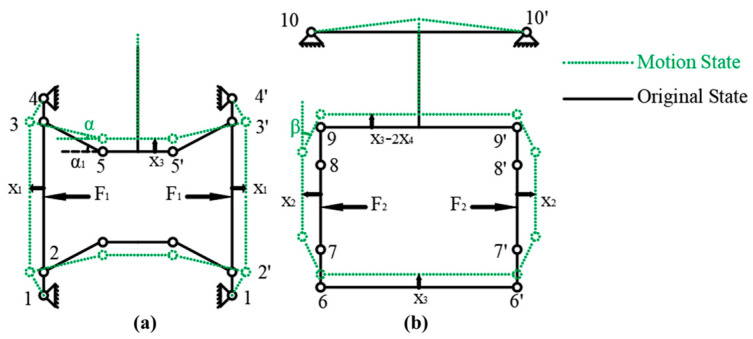
Kinematic models of the actuation stator. (**a**) The lower clamping mechanism. (**b**) The upper clamping mechanism.

As shown in Figure 6, the kinematic model of the actuation stator is derived based on the pseudo-rigid-body method with appropriate simplifications.

Therefore, the kinetic energy and potential energy equations of the actuation stator can be derived as:

(22) $$\left\{\begin{array}{cc} T= & \left[\frac{1}{2}J_{12-1}{\left(\frac{v_{1}}{l_{12}}\right)}^{2}\times 2+\frac{1}{2}m_{23}{v_{1}}^{2}\right]\times 2+\left[\frac{1}{2}\left(J_{35-O}+m_{35}{\left(\frac{l_{35}}{2}\right)}^{2}\right){\left(\frac{v_{1}}{l_{35}\sin\alpha }\right)}^{2}\right]\times 4\\ & +\frac{1}{2}m_{55^{\prime }66^{\prime }}{v_{3}}^{2}+\frac{1}{2}m_{55^{\prime }66^{\prime }}{v_{3}}^{2}+\frac{1}{2}m_{78}\left[{v_{2}}^{2}+{\left(v_{3}-v_{4}\right)}^{2}\right]\times 2+\frac{1}{2}m_{99}{\left(v_{3}-{2v}_{4}\right)}^{2}\\ & +4\times \frac{1}{2}{\times \left[\frac{\left(v_{3}-2v_{4}\right)sin\theta }{\cos\left(\theta +\beta \right)l_{89}}\right]}^{2}\left[J_{89-o}+m_{89}\frac{2{l_{89}}^{2}\times \left[{\left(\frac{\cos\left(\theta +\beta \right)}{sin\theta }\right)}^{2}+{\left(\frac{cos\beta }{sin\theta }\right)}^{2}-1\right]}{4}\right]\\ V= & \left[\frac{1}{2}k{\left(\frac{x_{1}}{l_{12}}\right)}^{2}\times 4+\frac{1}{2}k({\alpha_{1}-\alpha )}^{2}\times 2\right]\times 2+\frac{1}{2}k\beta^{2}\times 8+\frac{1}{2}k{\left(\frac{x_{3}-{2x}_{4}}{\frac{1}{2}l_{1010^{\prime }\;}}\right)}^{2}\times 2\\ v_{1}= & \dot{x_{1}},\;v_{2}=\dot{x_{2}},\;v_{3}=\dot{x_{3}},\;v_{4}=\dot{x_{4}}\\ v_{3}= & \frac{v_{1}}{\tan\alpha },{\;v}_{4}=v_{2}\tan\beta \\ x_{3}= & \frac{2lsin\;\alpha_{1}+\sqrt{4l^{2}\sin^{2}\alpha_{1}-4\left(x_{1}^{2}+2x_{1}lcos\;\alpha_{1}\;\right)}}{2}\\ x_{4}= & \frac{2l+\sqrt{{4l}^{2}-4x_{2}^{2}}}{2}\\ cos\alpha = & \frac{x_{1}+l_{35}\cos\alpha_{1}}{l_{35}}\\ sin\beta = & \frac{x_{2}}{l_{89}}\\ tan\theta = & \frac{v_{2}}{v_{3}-v_{4}} \end{array},\right.$$

where $J_{ij-k}$ represents the moment of inertia of the link between hinge i and hinge j, rotating about hinge *k*. $J_{ij-o}$ represents the moment of inertia of the link between hinge i and hinge j, rotating about its center of mass. $m_{ij}$ represents the mass of the link between hinge i and hinge $j.l_{ij}$ represents the length of this link.

By Lagrange’s equations, the kinematic models and the natural frequency expression are derived as:

(23) $$\left\{\begin{array}{cc} M= & \left[\begin{array}{cc} m_{11} & 0\\ 0 & m_{22} \end{array}\right]\\ K= & \left[\begin{array}{cc} \frac{8k}{l_{12}^{2}}+\frac{8k}{l_{1010^{\prime }}^{2}{tan}^{2}\alpha_{1}} & 0\\ 0 & \frac{8k}{l_{76}^{2}} \end{array}\right]\\ F= & \left[\begin{array}{c} F_{1}\\ F_{2} \end{array}\right]\\ \ddot{X}= & \left[\begin{array}{c} \ddot{x_{1}}\\ \ddot{x_{2}} \end{array}\right]\\ X= & \left[\begin{array}{c} x_{1}\\ x_{2} \end{array}\right]\\ m_{12}= & 2\times \left(\begin{array}{c} \frac{2J_{12-1}}{l_{12}^{2}}+m_{23}+\frac{J_{35-O}+m_{35}{\left(\frac{l_{35}}{2}\right)}^{2}}{{\left(l_{35}\sin\alpha_{1}\right)}^{2}}\times 2+\frac{m_{55^{\prime }66^{\prime }}+2m_{78}+m_{99}}{2{tan}^{2}\alpha_{1}}+2\frac{m_{98}}{{tan}^{2}\alpha_{1}} \end{array}\right)\\ m_{22}= & 2\times \left(\frac{2J_{89-o}}{l_{89}^{2}}+m_{78}\right) \end{array}\right.,$$

(24) $$f=\frac{1}{2\pi }\sqrt{\frac{K}{M}}.$$

where *K* represents the mass matrix of the actuation stator. *M* represents the stiffness matrix of the actuation stator.

## 4. Finite Element Analysis and Evaluation

### 4.1. Finite Element Analysis of Actuation Stator Motion Characteristics

**Figure 7:**
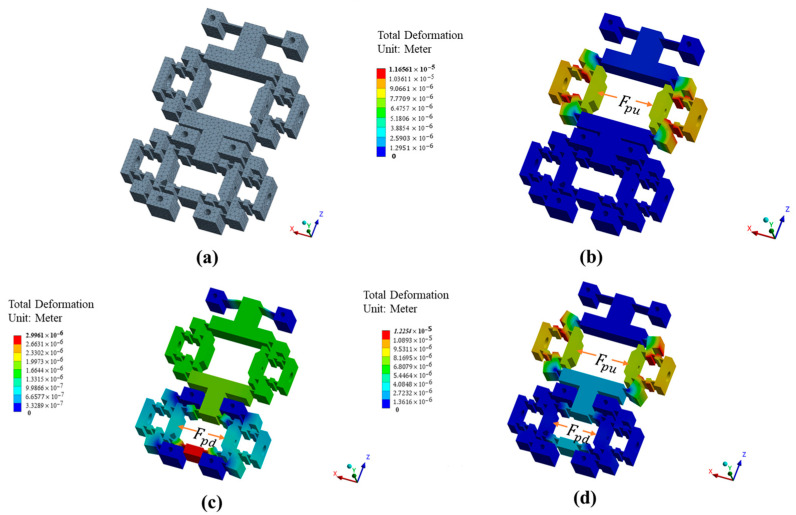
FEA of static properties for the actuation stator. (**a**) Mesh generation. (**b**) The load application conditions for the performance test of the upper clamping mechanism. (**c**) The load application conditions for the performance test of the lower clamping and driving mechanisms. (**d**) The load application conditions for the coupling rate test.

Ideally, the output of one clamping mechanism does not affect the output of the other clamping mechanism or the driving mechanism, and vice versa. To test the static properties of the actuation stator—including the output performance of the upper clamping mechanism, the output performance of the lower clamping mechanism, the output performance of the driving mechanism, and the decoupling characteristics between these mechanisms—finite element analysis tests are conducted, as shown in Figure 7. Input forces ranging from 0 N to 100 N are individually applied to evaluate the static properties. The results are shown in Table 2. $x_{mu}\;\mathrm{and}\;x_{md}$ respectively represent the output displacement along the x-axis at the end-effectors of the upper and lower clamping mechanisms, while $z_{m}$ represents the z-axis output displacement at the driving mechanism’s end-effector. $R_{mu-md}$ represents the coupling rate between $x_{mu}$ and $x_{md}$; $R_{mu-m}$ represents the coupling rate between $x_{mu}$ and $y_{m}$; and $k_{mu},k_{md}\;\mathrm{and}\;k_{mf}$ respectively represent the stiffness of the upper/lower clamping mechanisms and the driving mechanism.

Through the data in Table 2, the following conclusions can be drawn:

1. When only $F_{pu}$ is applied, as shown in Figure 7b, the upper clamping mechanism generates corresponding displacement, with minimal impact on the lower clamping mechanism and the driving mechanism; the coupling rates $R_{mu-md}$ and $R_{mu-m}$ reach only 0% and 7%, respectively. With the increase in $F_{pu}$, the target displacement $x_{mu}$ grows under constant ratios governed by $k_{mu}$, which remains at 2.18 constantly, validating the linear relationship between input force and output displacement in the upper clamping mechanism.
2. When only $F_{pd}$ is applied, as shown in Figure 7c, the lower clamping mechanism and the driving mechanism generate corresponding displacement, with minimal impact on the upper clamping mechanism; the coupling rates $R_{mu-md}$ and $R_{mu-m}$ reach only 6% and 2%, respectively. With the increase in $F_{pd}$, the target displacements $x_{mu}\;\text{and}\;z_{m}$ grow under constant ratios governed by $k_{md}\;\text{and}\;k_{mf}$, which remain at 26.72 and 10.91 constantly, validating the linear relationship between input force and output displacement in the lower clamping mechanism and driving mechanism.
3. When both $F_{pu}$ and $F_{pd}$ are applied, as shown in Figure 7d, the mechanism stiffness of the actuation stator undergoes slight variations. Within the input force range of 0–60 N, the stiffness of the upper clamping mechanism increases from 2.15 to 2.19, while the lower clamping mechanism exhibits a stiffness variation of 25.91–26.92. The driving mechanism demonstrates relatively more pronounced stiffness change, ranging from 7.74 to 11.45. Overall, the stiffness interactions between mechanisms due to motion are mutually influential but negligible in practical terms.

All in all, with the increase in driving force, all the corresponding displacements grow under constant ratios governed by $k_{mu},\;k_{md},\;\mathrm{and}\;k_{mf}$, which demonstrates that the actuation stator exhibits excellent linear output capability. Furthermore, the coupling rates remain below 7%, demonstrating satisfactory decoupling performance in the designed driving mechanism.

**Figure 8:**
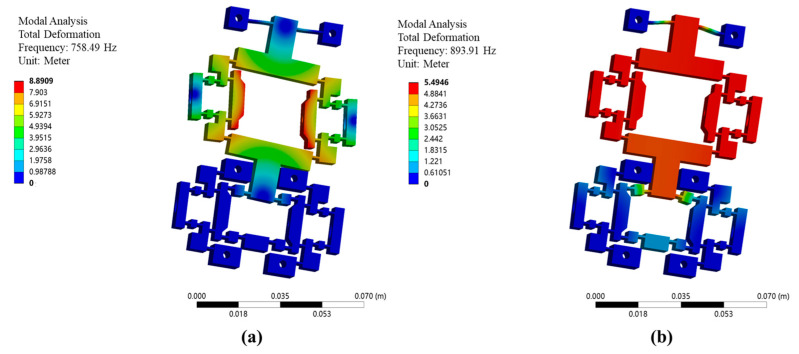
The natural frequency of the actuation stator test. (**a**) First-order natural frequency. (**b**) Second-order natural frequency.

The natural frequencies are validated using the finite element modal analysis module. The first-order natural frequency in the x-direction is measured as 758.49 Hz, as shown in Figure 8a, and the second-order natural frequency in the y-direction is measured as 893.91 Hz, as shown in Figure 8b. The relatively high natural frequencies of the actuation stator indicate a rational structural design.

### 4.2. Finite Element Analysis of Inchworm Piezoelectric Motor Characteristics

**Figure 9:**
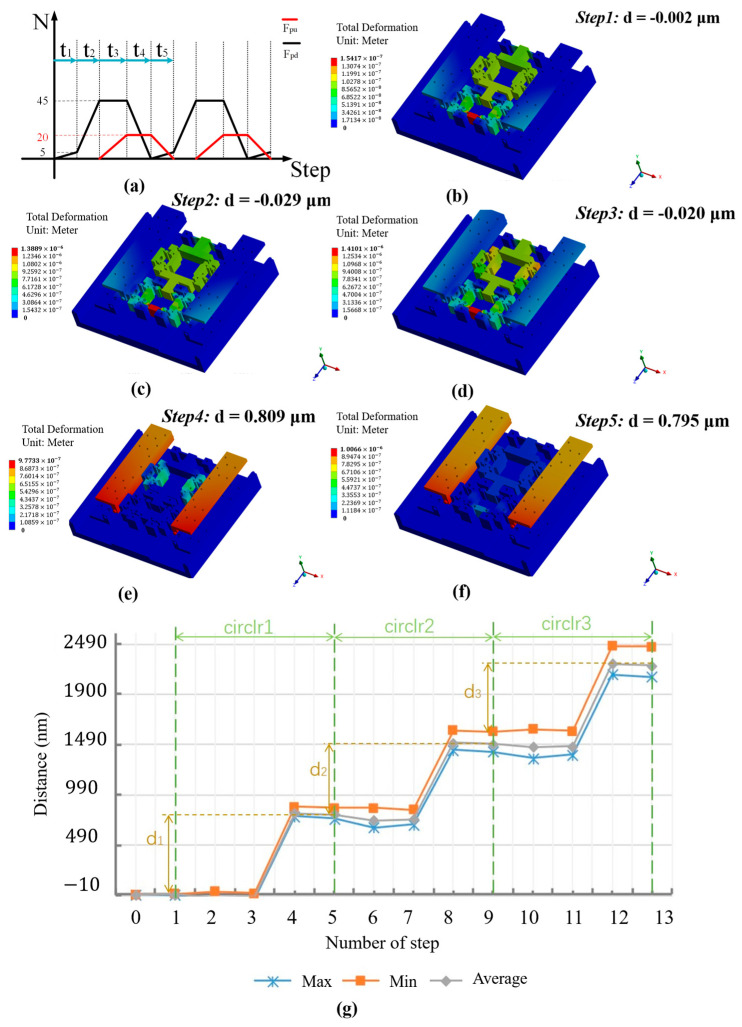
FEA of motion performance for the inchworm piezoelectric motor. (**a**) The excitation signal sequence of the input force. (**b**–**f**) FEA of motion generation. (**g**) Displacement of the inchworm piezoelectric motor in FEA.

To validate the motion characteristics of the inchworm piezoelectric motor, a finite element dynamic simulation is designed for the motor. First, the overall structure of the inchworm piezoelectric motor is simplified by omitting certain crossed roller guide rail components. The model is imported into the transient analysis module, and driving forces $F_{pu}$ and $F_{pd}$ are applied according to Figure 9a. As shown in Figure 9b–f, the displacement of the upper sliding stage at phases t0–t5 is obtained. (For clarity in visualizing the deformation of the actuation stator, the upper base plate is hidden. The displacement of the upper sliding stage can be observed by the upper preload baffle.) Three timing cycles are configured, and the displacement variation curve is shown in Figure 9g. The designed inchworm piezoelectric motor aligns well with the displacement generation principles derived from theoretical analysis. The backstep effect is almost completely eliminated, and the stepping displacement remains stable. Under the configured timing forces, each cycle generates a step distance of d=0.797 μm.

### 4.3. Finite Element Analysis of Actuation Stator Fatigue Life

**Figure 10:**
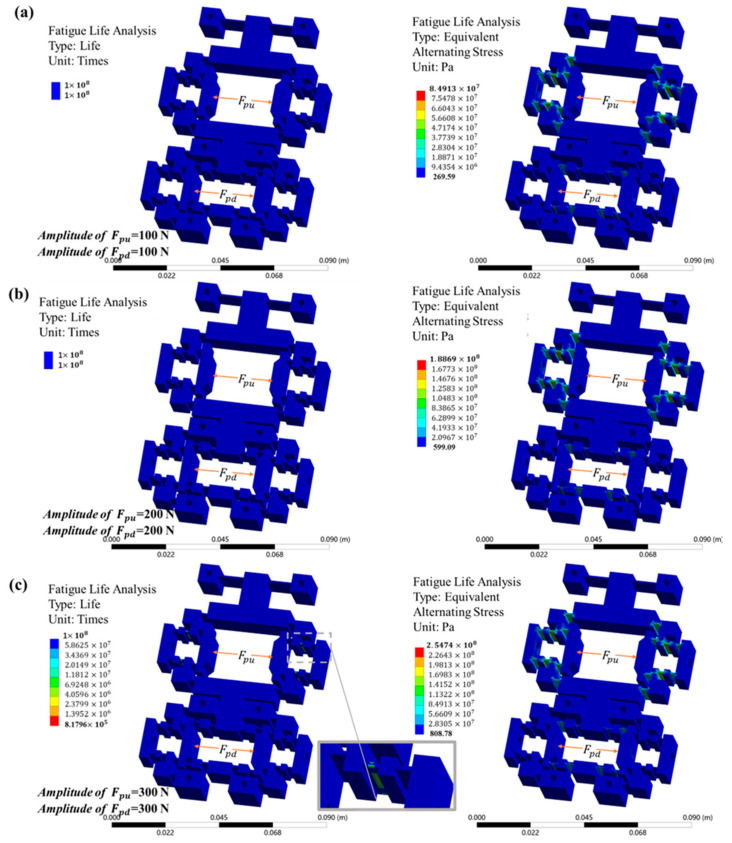
Fatigue life test of the actuation stator: distribution of life and alternating stress under sinusoidal alternating load conditions with amplitudes of (**a**) 100 N, (**b**) 200 N, and (**c**) 300 N.

In the aforementioned simulation analysis, the maximum input force applied to the actuation stator was 45 N. To evaluate its fatigue performance under the designed structural and loading conditions based on the principle of conservative design, fatigue simulation tests were conducted on the actuation stator by applying sinusoidal alternating loads at elevated amplitudes of 100 N, 200 N, and 300 N. The fatigue life and equivalent alternating stress of the structure under these different sinusoidal loading conditions were analyzed, and the results are shown in Figure 10.

In engineering practice, a fatigue life exceeding 10^7^ cycles is characteristic of high-cycle fatigue behavior, which demonstrates the structure’s retained fatigue resistance sufficient to ensure long-term integrity under such loading conditions. Accordingly, regions exhibiting fatigue lives exceeding 10^8^ cycles are classified within the 10^8^-cycle category throughout this study. The results showed that, under the tests of 100 N and 200 N beyond the maximum input force, the fatigue performance of the stage could meet the requirements. Furthermore, under the 300 N input force test, there was a localized occurrence of stress concentration. Specifically, the hinge area in the upper clamping mechanism is the critical region for fatigue crack initiation and exhibits the shortest lifespan among all structural components due to its relatively thin local thickness and high strain level, as shown in Figure 10c. It is noteworthy that, even under 300 N input force, this region’s fatigue life exceeds 10^6^ cycles, confirming the actuation stator’s durability compliance within intended operating conditions. However, as the load increases, the service life of the actuation stator exhibits a noticeable decrease. Under high-load conditions, careful attention should be paid to its operating duration and frequency.

## 5. Experimental Results

**Figure 11:**
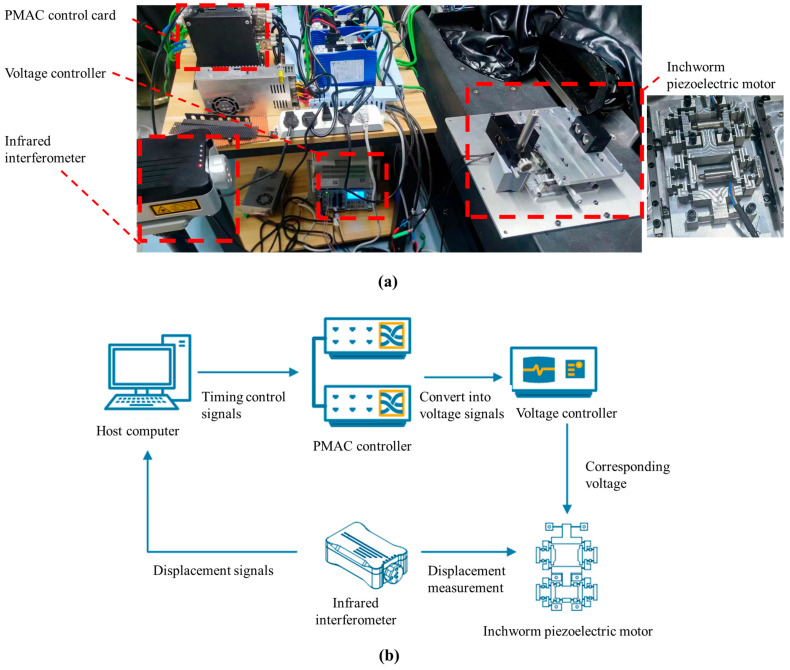
Experimental setup. (**a**) The prototype testing system. (**b**) The hardware configuration.

In this section, the motion performance of the inchworm piezoelectric motor is tested by constructing a dedicated experimental setup. The prototype testing system and hardware configuration are shown in Figure 11. Two piezoelectric actuators (PSt150/5/7VS10) were selected to drive the motion motor. The motion strategy was implemented by using a Power PMAC controller and developed with the C programming language. In the experiment, the environmental temperature was controlled within 0.02 °C by the constant temperature and humidity equipment. A Renishaw laser interferometer was used to examine the motion performance of the stage.

Based on the simulation results above, the operating frequency of the piezoelectric motor was set to a range of 0–750 Hz. Three sets of maximum voltage amplitude combinations were applied to piezoelectric actuators s1 and s2: 135 V + 60 V, 67.5 V + 30 V, and 33.75 V + 15 V. The displacement, velocity, and load capacity of the piezoelectric motor were measured under these conditions.

**Figure 12:**
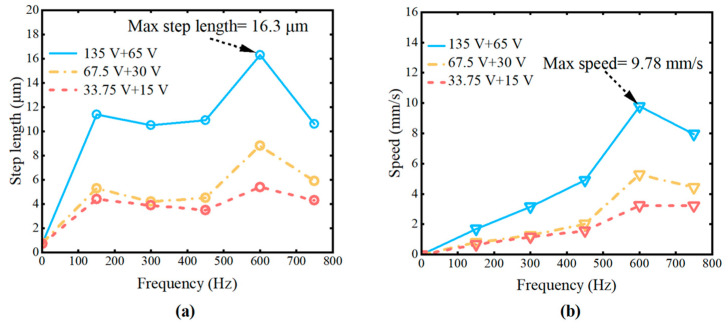
The motion performance test of the inchworm piezoelectric motor. (**a**) Step length at varying frequencies. (**b**) Speed at varying frequencies.

As shown in Figure 12a,b, the following conclusions can be derived from the experimental results:

1. Under the same operating frequency, the displacement of the piezoelectric motor increases with higher operating voltage;
2. Under the same operating frequency, the motion velocity increases with higher operating voltage;
3. At the same operating voltage, the motion velocity increases with higher operating frequency;
4. When the frequency is 600 Hz and the input voltage is set to 135 V + 65 V, the maximum step size of 16.3 μm and the maximum velocity of 9.78  mm/s  are achieved.

**Figure 13:**
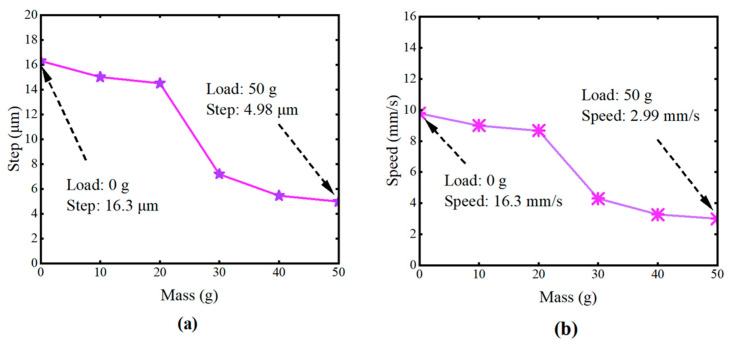
Load capacity test of the inchworm piezoelectric motor. (**a**) Step length varying mass load. (**b**) Step length varying mass load.

To evaluate the load capacity of the piezoelectric motor, a driving frequency of 600 Hz and operating voltages of 135 V + 65 V were selected. As shown in Figure 13a,b, displacement and velocity curves under loads ranging from 0 to 50 g were tested. Under loads of 40–50 g, the motor’s displacement and velocity gradually stabilize. Overall, the piezoelectric motor demonstrates acceptable load capacity under these conditions.

## 6. Conclusions

In this study, a novel high-precision inchworm-type piezoelectric motor is developed for achieving accurate positioning operation in the field of wafer-level electron beam inspection. The structure design and corresponding working principle are presented in detail. Specifically, an actuation stator with two clamping mechanisms and two piezoelectric elements is designed. Considering the characteristics of this stator, a series of excitation signal sequences is proposed to determine the alternating motions of the upper/lower clamping mechanisms for generating stepping linear displacement and mitigating backstep effects. In the theoretical section, the actuation stator is analyzed by using the compliance matrix method to derive its output compliance, input stiffness, and mechanical amplification ratio. Finite element analysis is also employed for model validation and performance evaluation based on the theoretical analysis. The experimental setup is fabricated and established to carry out the verification. Under a driving frequency of 600 Hz, the piezoelectric motor achieved a maximum step size of 16.3 μm and a maximum speed of 9.78 mm/s. Under various loads, the motor also can provide precise actuation. It was confirmed that the proposed piezoelectric motor can achieve superior motion resolution, operational stability, and acceptable load capacity. This is especially useful for the wafer-level electron beam inspection system to achieve precision motion positioning operation. In the future, we will investigate influence of temperature changes on the performance of the stage.

## Figures and Tables

**Table 1:** The material properties of AL 7075-T651.

Material	Elastic Modulus (GPa)	Yield Strength (MPa)	Poisson’s Ratio	Density (kg/m^3^)
AL7075-T651	71.7	505	0.33	2180

**Table 2:** The finite element analysis experimental data.

$F_{pu}/N$	$F_{pd}/N$	$x_{mu}/$μm	$x_{md}/$μm	$z_{m}/$μm	$R_{mu-md}$	$R_{mu-m}$	$k_{mu}$	$k_{\mu d}$	$k_{mf}$
20.00	0.00	9.18	0.04	0.62	0.00	0.07	2.18	-	-
40.00	0.00	18.36	0.08	1.25	0.00	0.07	2.18	-	-
60.00	0.00	27.53	0.12	1.87	0.00	0.07	2.18	-	-
80.00	0.00	36.71	0.16	2.50	0.00	0.07	2.18	-	-
100.00	0.00	45.89	0.21	3.12	0.00	0.07	2.18	-	-
0.00	20.00	0.04	0.75	1.83	0.06	0.02	-	26.72	10.91
0.00	40.00	0.09	1.50	3.67	0.06	0.02	-	26.72	10.91
0.00	60.00	0.13	2.25	5.50	0.06	0.02	-	26.72	10.91
0.00	80.00	0.18	2.99	7.33	0.06	0.02	-	26.72	10.91
0.00	100.00	0.22	3.74	9.17	0.06	0.02	-	26.72	10.91
20.00	20.00	9.26	0.77	2.46	-	-	2.16	25.90	8.13
20.00	40.00	9.29	1.49	4.29	-	-	2.15	26.92	9.33
20.00	60.00	9.19	2.30	5.24	-	-	2.18	26.13	11.45
40.00	20.00	18.31	0.79	2.48	-	-	2.18	25.41	8.05
40.00	40.00	18.27	1.57	4.39	-	-	2.19	25.43	9.12
40.00	60.00	18.23	2.31	5.40	-	-	2.19	26.03	11.12
60.00	20.00	27.47	0.77	2.58	-	-	2.18	25.91	7.74
60.00	40.00	27.42	1.53	4.50	-	-	2.19	26.15	8.89
60.00	60.00	27.35	2.28	5.57	-	-	2.19	26.29	10.78

## Data Availability

The original contributions presented in the study are included in the article; further inquiries can be directed to the authors.
